# Variation in migration pattern, broodstock origin, and family productivity of coho salmon hatchery populations in British Columbia, Canada, derived from parentage‐based tagging

**DOI:** 10.1002/ece3.5530

**Published:** 2019-08-18

**Authors:** Terry D. Beacham, Colin Wallace, Kim Jonsen, Brenda McIntosh, John R. Candy, David Willis, Cheryl Lynch, Ruth E. Withler

**Affiliations:** ^1^ Fisheries and Oceans Canada Pacific Biological Station Nanaimo BC Canada; ^2^ Fisheries and Oceans Canada Regional Headquarters Vancouver BC Canada

**Keywords:** coded‐wire tags, coho salmon, fishery management, genetic stock identification, genotyping by sequencing, parentage‐based tagging, sibs

## Abstract

In salmonid parentage‐based tagging (PBT) applications, entire hatchery broodstocks are genotyped, and subsequently, progeny can be nonlethally sampled and assigned back to their parents using parentage analysis, thus identifying their hatchery of origin and brood year (i.e., age). Inter‐ and intrapopulation variability in migration patterns, life history traits, and fishery contributions can be determined from PBT analysis of samples derived from both fisheries and escapements (portion of a salmon population that does not get caught in fisheries and returns to its natal river to spawn). In the current study of southern British Columbia coho salmon (*Oncorhynchus kisutch*) populations, PBT analysis provided novel information on intrapopulation heterogeneity among males in the total number of progeny identified in fisheries and escapements, the proportion of progeny sampled from fisheries versus escapement, the proportion of two‐year‐old progeny (jacks) produced, and the within‐season return time of progeny. Fishery recoveries of coho salmon revealed heterogeneity in migration patterns among and within populations, with recoveries from north and central coast fisheries distinguishing “northern migrating” from “resident” populations. In northern migrating populations, the mean distance between fishery captures of sibs (brothers and sisters) was significantly less than the mean distance between nonsibs, indicating the possible presence of intrapopulation genetic heterogeneity for migration pattern. Variation among populations in productivity and within populations in fish catchability indicated that population selection and broodstock management can be implemented to optimize harvest benefits from hatcheries. Application of PBT provided valuable information for assessment and management of hatchery‐origin coho salmon in British Columbia.

## 1 INTRODUCTION

Life history variability in salmonids, generally under both genetic and environmental control, is known to be condition‐dependent with the adoption of differing developmental pathways affected by a myriad of factors such as individual size, season, age, predation, and food availability. This variation among and within populations not only contributes to resilience and persistence in salmonids (Schindler et al., 2010), but also affects the extent and pattern of harvest they experience (Kendall & Quinn, 2009, 2011). Life history variation in hatchery‐supplemented salmon populations propagated primarily for harvest augmentation will influence the degree to which they beneficially contribute to harvest or detrimentally escape capture and interact with natural‐origin fish in river spawning locations (Davison & Sattherwaite, 2017). Evaluation of populations for hatchery supplementation and management of associated fisheries can therefore be facilitated with accurate information on inter‐ and intrapopulation variation in traits such as age of maturity, spatial and temporal extent of migration patterns, spawner productivity, straying tendency, and the likelihood of capture versus avoidance of fishery gear.

Coho salmon (*Oncorhynchus kisutch*) have been supplemented with hatchery propagation in British Columbia (BC) and adjoining American jurisdictions for over 40 years to increase ocean harvest and supplement populations of conservation concern. All coho salmon released since the late 1990s from most large hatcheries in southern British Columbia, Washington, and Oregon have been visually marked with an adipose fin clip to facilitate mark‐selective fisheries intended to target hatchery salmon and spare naturally spawned (unclipped) individuals. Not only does the presence of life history variation in hatchery fish impact the economic benefit derived from their harvest, but also selective harvest practices can in turn lead to altered life history and phenology in exploited populations (Hard et al., 2008; Tillotson & Quinn, 2018). Therefore, evaluation of life history variation among and within hatchery populations can facilitate both selection of populations well suited for harvest augmentation and evaluation of the subsequent impacts of exploitation rates on the populations under enhancement. Management of both genetic (broodstock selection and spawning protocols) and environmental (spawn timing, rearing conditions, ration levels) factors to affect hatchery fish characteristics and distributions and increase their utility may be possible.

Variation in migration trajectory and distance is a primary feature of intraspecific life history diversity in Pacific salmonids. The rich feeding grounds of marine waters generally fuel the rapid growth required for reproduction in these organisms, but come at the cost of high energy requirements for smoltification (the transition from osmoregulation in freshwater to saltwater) and migration as well as a possibly reduced survival level due to predation. Previous examination of eastern Pacific salmonid migration has indicated that juveniles move primarily northward upon ocean entry, following the continental shelf in a northwest direction (Fisher et al., 2007; Hartt & Dell, 1986; Tucker et al., 2009, 2012). However, coded‐wire tag recovery from fishery catches indicated that adult coho salmon from different freshwater regions inhabited different areas of the coastal ocean and likely undertook spatial differentiation earlier in ocean residence. The observed differences in distribution were suggested to reflect a significant level of interpopulation genetic differentiation (Weitkamp, 2011; Weitkamp & Neely, 2002). Morris et al. (2007) documented that individual coho salmon populations can be composed of two migratory components, a fast component that undertakes rapid and direct northwest migration upon entering the ocean, and a slow component that migrates a relatively short distance from the natal river and takes up winter residence over the continental shelf.

Recent detailed investigations of early marine distribution in coho salmon based on tagging indicated that inter‐ and intrapopulation coho salmon distribution is more complex than a simple dichotomy of long and short migration routes overlaying interpopulation differentiation in migration trajectory. Some coho salmon originating from watersheds that discharge into the Salish Sea (inside waters of Puget Sound and the Strait of Georgia) undertook residency within the Salish Sea, with a further subset of the residents embarking on subsequent migratory excursions to outside coastal waters, presumably for feeding opportunity (Rohde, Fresh, & Quinn, 2014; Rohde, Kagley, Fresh, Goetz, & Quinn, 2013). This work revealed that both partial migration, in which only a portion of a population is migratory, and differential migration, in which migratory individuals undertake journeys of variable distance, are features of coho salmon life history variation.

The discovery of significant intrapopulation diversity in migration patterns in coho salmon is consistent with observations for other salmonid life history traits, which vary both among and within populations. Of interest is whether the intrapopulation diversity in migration and other traits reflects family differentiation, as would be expected if it arises primarily from genetic heterogeneity and/or environmental conditions common to family members (e.g., spawning date and associated incubation time, temperature, emergence size traits). A family‐specific influence on migration would indicate that selection of broodstock, spawn timing, mating protocols, and incubation and rearing conditions within the hatchery might all influence the resulting migratory tendencies of the hatchery progeny. Whereas physical tagging and genetic stock identification techniques have led to the current level of understanding of migratory and other diversity in coho salmon (Beacham et al., 2016; Morris et al., 2007), a new methodology is required for detailed analysis of intrapopulation diversity.

Parentage‐based tagging (PBT) in salmonids entails the genotyping of parental fish, typically the entire broodstock of one or more hatcheries, to enable the subsequent assignment of lethally and nonlethally sampled progeny back to their parents within the hatchery broodstocks (Anderson & Garza, 2006; Steele, Hess, Narum, & Campbell, 2019). Assignment of progeny to parents through standard exclusion or probability‐based methods provides the age as well as the hatchery and family of origin for progeny sampled at any location or time throughout their lives. PBT techniques developed and validated for a number of Pacific salmonids are increasingly being used to provide comprehensive identification and assessment objectives (Beacham et al., 2017, 2018; Hess et al., 2016; Steele et al., 2019). Beacham et al. (2019) demonstrated the utility of PBT for the aging and identification of southern BC hatchery coho salmon in highly mixed‐stock fisheries throughout BC and in adult returns to rivers known as escapements (portion of a salmon population that does not get caught in fisheries and returns to its natal river to spawn). In the current study, we use coho salmon PBT analysis based on genotyping of 304 variable SNPs to examine life history variation among and within hatchery coho salmon populations, with a focus on family differentiation in productivity and fishery contributions.

PBT was applied to coho salmon sampled from fisheries and escapements (including both hatchery broodstock and hatchery‐origin individuals spawning in natural environments) in BC. A primary objective of this study was to examine spatial and temporal variability in migration patterns among hatchery coho salmon populations in southern BC based on PBT identifications in fishery catches. Additionally, we evaluated intrapopulation heterogeneity among male spawners in the total number of progeny contributed to fisheries and escapements, the proportion of progeny sampled from fisheries versus escapement, and the proportion of two‐year‐old male and female progeny (jacks, jills) produced. We examined the paternal‐progeny within‐season return time relationship in hatchery escapement data. Complete genotyping of 20 hatchery broodstocks in 2014 provided a parental database of over 6,000 individuals. Commercial and recreational coho salmon fisheries were sampled in 2017, and hatchery broodstock and adipose fin‐clipped individuals in river escapements were sampled in 2016 and 2017 to identify progeny contributions from the 2014 hatchery broodstock parents. A total of 21,195 individuals were genotyped from fishery, hatchery brood, and escapement sampling, and PBT was used to identify as many individuals as possible to hatchery and parents of origin.

Analysis of the data provided insight into population‐specific distributions among fisheries, the 2014 and 2015 hatchery parental contributions to 2017 hatchery broodstocks and associated stray rates among populations, and productivity of specific return time components of hatchery broodstocks. Fine‐scale geographic variability among populations in fishery distribution and timing of catch indicated that individual hatcheries varied significantly not only in overall contribution to fisheries but also by harvest location. We conclude that PBT analysis increases the scope for hatchery broodstock and fishery assessment to improve hatchery broodstock management for improved harvest contributions and reduced impact on natural populations.

## 2 METHODS

### 2.1 Fishery sample collection

**Figure 1:**
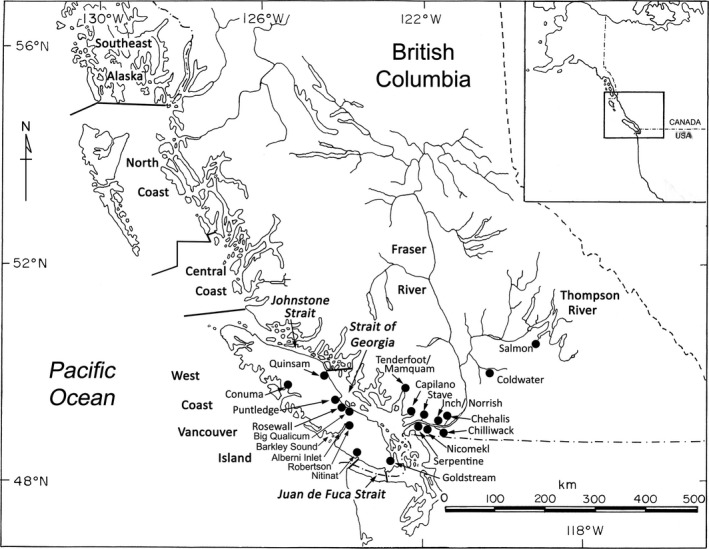
Map indicating geographic locations for fishery sampling and 20 populations for which parentage‐based tagging was applied in estimation of stock composition

Six fishery areas were defined for coho salmon sampled from fisheries conducted in BC during 2017. The fishery areas were as follows: North Coast (NC), Central Coast (CC), Johnstone Strait (JS), Strait of Georgia (SOG), Juan de Fuca Strait (JDF), and west coast of Vancouver Island (WCVI) (Figure 1). Samples from commercial, recreational, and First Nations fisheries within a fishery area were pooled, and samples from Barkley Sound and Alberni Inlet were pooled with WCVI samples. Further details on fishery sampling were outlined by Beacham et al. (2019).

### 2.2 Escapement and broodstock sample collection

Twenty hatchery broodstocks comprising 6,061 individuals were genotyped in 2014 (Beacham et al., 2017). The hatchery broodstocks originated from the lower Fraser River, southern BC mainland, and Vancouver Island, and constituted the main parental base for subsequent PBT assignments in 2016 and 2017 fishery and escapement sampling. The same 20 hatchery broodstocks were again genotyped in 2015, allowing for potential identification of jacks or jills (age 2 years males or females, respectively) in the 2017 hatchery broodstock sampling.

In 2016, adipose fin‐clipped jacks were sampled from nonbroodstock escapement as outlined by Beacham et al. (2019) to evaluate accuracy of assignments of the jacks, under the assumption of no straying among populations for the individuals sampled. Additionally, individuals in the 2016 hatchery broodstocks were genotyped to identify age‐2 fish originating from 2014 broodstock parents by PBT. A total of 7,219 brood and nonbrood fish from 2016 escapements were genotyped.

In 2017, escapements (nonbroodstock hatchery and river returns, clipped individuals only) from 13 populations (1,692 individuals) were sampled with the objective of evaluating stray rates among populations (Beacham et al., 2019). In addition, 6,002 broodstock were genotyped from the same 20 hatchery populations, except that the Goldstream River broodstock was not sampled in 2017, precluding analyses of variation in productivity for this population.

### 2.3 Genotyping

The detailed procedure for library preparation and genotyping was outlined by Beacham et al. (2017), and a summarized version was provided by Beacham et al. (2019). The process involved loading amplified DNA from 756 individuals (up to 304 amplicons per individual) on a P1 chip v3 (chip used with the Ion Torrent Proton sequencer) with an Ion Chef (laboratory instrument used to robotically load DNA libraries on to a sequencing chip). Two chips were loaded consecutively with one run of the Ion Chef, both chips were then subsequently loaded onto an Ion Torrent Proton sequencer, and the genotype of each individual recorded with automated scoring of the genotype via Proton software Variant Caller® at one SNP site in each amplicon. Genotypes at all available SNPs for each individual were assembled to provide multilocus genotypes that were the basic input for PBT analysis.

### 2.4 Identification of individuals

PBT was used to identify individuals in fishery and escapement samples by matching the genotype of the individual to the genotypes of prospective parents via the COLONY software package (Jones & Wang, 2010; Wang, 2016). COLONY was utilized as it can produce assignments when the genotype of one of the parents is missing, either due to a missing parental sample or due to failure to produce a parental genotype from an existing sample. Given that PBT assignments for 20 potential populations were evaluated for each fishery and escapement sample, COLONY was run with all broodstock sampled during 2014 input as a single unit for analysis of fishery and escapement samples, with no differentiation among populations. Although the COLONY assumption of a single population in the parent pool was violated, analysis of known‐origin samples indicated that very high levels of accuracy were achieved in assignments when pooling of potential parents in contributing populations was conducted (Beacham et al., 2019). Two‐parent assignments were accepted only when both assigned parents originated from the same population. Two‐parent and single‐parent assignments were accepted only when the probability of correct assignment was ≥0.85 for the parent pair or single parent. Polygamous mating was assumed for the COLONY analysis. Individuals with more than 120 missing genotypes were eliminated from further analyses. An estimated genotyping error rate of 1% was used for COLONY assignments. Previously, Beacham et al. (2017) had reported that an average genotyping error rate of 1.07% (1,220 discrepancies in 114,105 comparisons) or an allele error rate of 0.53% (1,220 discrepancies in 228,210 comparisons) was observed over the 304 SNPs scored. The parent pair output file was the basic file used in subsequent analyses.

The baseline for individuals sampled in the 2016 escapements and 2017 fisheries and escapements included all broodstocks sampled in 2014 and 2015, as coho salmon in southern BC are predominately three years of age (Sandercock, 1991). Only age‐two jacks were identified in the 2016 escapement sampling, whereas both jacks and age‐three fish were identified in the 2017 escapements. Jacks were typically visually identified by hatchery staff based on small body size.

### 2.5 Estimation of distance between fishery areas

The approximate geographic midpoint of each fishery area was estimated, and the corresponding latitude and longitude was determined via http://www.mapdevelopers.com/geocode_tool.php. The water distance between points referenced by latitude and longitude was determined via http://www.csgnetwork.com/gpsdistcalc.html. Reference points for distance calculations between fishery areas were as follows: 54.32N, 131.67W (NC), 51.67N, 128.42W (CC), 50.48N, 126.33W (JS), 49.42N, 124.10W (SOG), 48.27N, 123.39W (JDF), and 49.00N, 126.13W (WCVI). In order to obtain representative water distances between areas, we defined two additional points, one at the north end of Vancouver Island (50.99N, 128.44W) and one at the south end (48.81N, 123.00W). Distances between the CC fishery area and southern fishery areas were determined by first calculating the distance between the CC location and the northern Vancouver Island location, and then adding the water distance between the northern Vancouver Island location and either the WCVI or JS location. The distance between the fishery areas was calculated as the sum of the water distance measures. Similarly, the distance between the SOG and JDF areas was calculated by first determining the water distance between the fishery location and the location at the southern end of Vancouver Island, and adding to it the water distance between the southern location and the respective fishery area. The matrix of water distances between fishery areas is outlined in Table 1.

**Table 1:** Distance matrix (km) between fishery areas

Fishery	CC	JS	SOG	JDF	WCVI
NC	366	601	800	935	718
CC	0	235	434	569	352
JS		0	199	371	435
SOG			0	172	389
JDF				0	217

Note

Fishery areas are as follows: NC, North Coast; CC, Central Coast; JS, Johnstone Strait; SOG, Strait of Georgia; JDF, Juan de Fuca Strait; WCVI, west coast Vancouver Island.

### 2.6 Comparison of fishery distances between sibs and nonsibs

The PBT marine fishery assignments outlined by Beacham et al. (2019) were used in the estimation of distance between fishery areas for sib and nonsib progeny. Only progeny assigned to both parents were used, providing 1,119 individuals for analysis. For marine fishery recoveries within a population, individuals sharing a common father were defined as sibs. The typical hatchery spawning design was to cross a single male with a single female, but in practice, some males produced offspring from more than one female, presumably as a result of carryover of viable sperm in a fertilization bucket even with intervening rinsing and drying between fertilizations. The distances between fishery recoveries (Table 1) of all pairwise combinations of the sibs were tabulated, as were the distances between all pairwise combinations of all nonsibs for each population. The data from three populations were subsequently eliminated from further analysis. There were no recoveries of sibs for two populations (Puntledge River, Rosewall Creek), and all recoveries for the Nicomekl River population were obtained from a single fishery (JDF). This reduced the analysis to 1,101 individuals.

Populations were sorted into two groups, termed northern migrating and resident. Northern migrating populations had at least 5% of total recoveries from the NC and CC fishery areas, and resident populations had fishery recoveries almost exclusively from southern BC. Sib and nonsib fishery distance recoveries were pooled over all populations within each group, and an ANOVA (R Core Team, 2019) was used to evaluate whether fishery recovery distances between sibs were less than those between nonsibs in each group.

### 2.7 Origins of 2017 hatchery broodstocks

PBT was used to assign adult and jack individuals in 19 hatchery broodstocks sampled in 2017 to parents in the 2014 or 2015 hatchery broodstocks. Strays were identified via PBT assignment to populations other than the one in which they were sampled in 2017.

### 2.8 Productivity of males

Progeny contributions by individual male spawners to 2017 fisheries, and 2016 and 2017 hatchery broodstock and nonbroodstock escapements were determined. These contributions included progeny sampled from 2017 marine and freshwater fisheries and jacks and adults sampled from the 2016 and 2017 escapement, respectively. As the Goldstream River broodstock was not sampled or genotyped in 2017, productivity of male spawners will be underestimated for this population. Individual males were investigated for a propensity to contribute disproportionately to fisheries, jack returns, or escapement relative to the average male contributions of the population. Deviations from expected progeny distributions within families relative to total family PBT identifications were evaluated by comparison with the observed population distribution by Fisher's exact test (Fisher, 1954). The analysis was restricted to males with at least four progeny sampled.

### 2.9 Return and spawn time

Of the 20 hatchery broodstocks surveyed in 2014, only the Chilliwack River and Capilano River populations returned to spawn or spawned over at least a three‐month period. The Chilliwack River broodstock was spawned from October through December in 2014. At the Capilano River, individuals returning from early April to June were defined as Early by hatchery staff, those returning from July through September were defined as Mid, and those returning from October through January were defined as Late. Total, fishery, and escapement progeny per spawning male were summarized by spawning month for the Chilliwack River broodstock and by the Early, Mid, and Late categories for the Capilano River hatchery broodstock. Differences in the distribution of progeny among spawning groups were evaluated in regional and monthly fisheries with Fisher's exact test. Differences in total and escapement progeny recoveries among spawning groups within a populations were evaluated for statistical significance with an ANOVA.

## 3 RESULTS

### 3.1 Sib versus nonsib marine fishery captures

**Figure 2:**
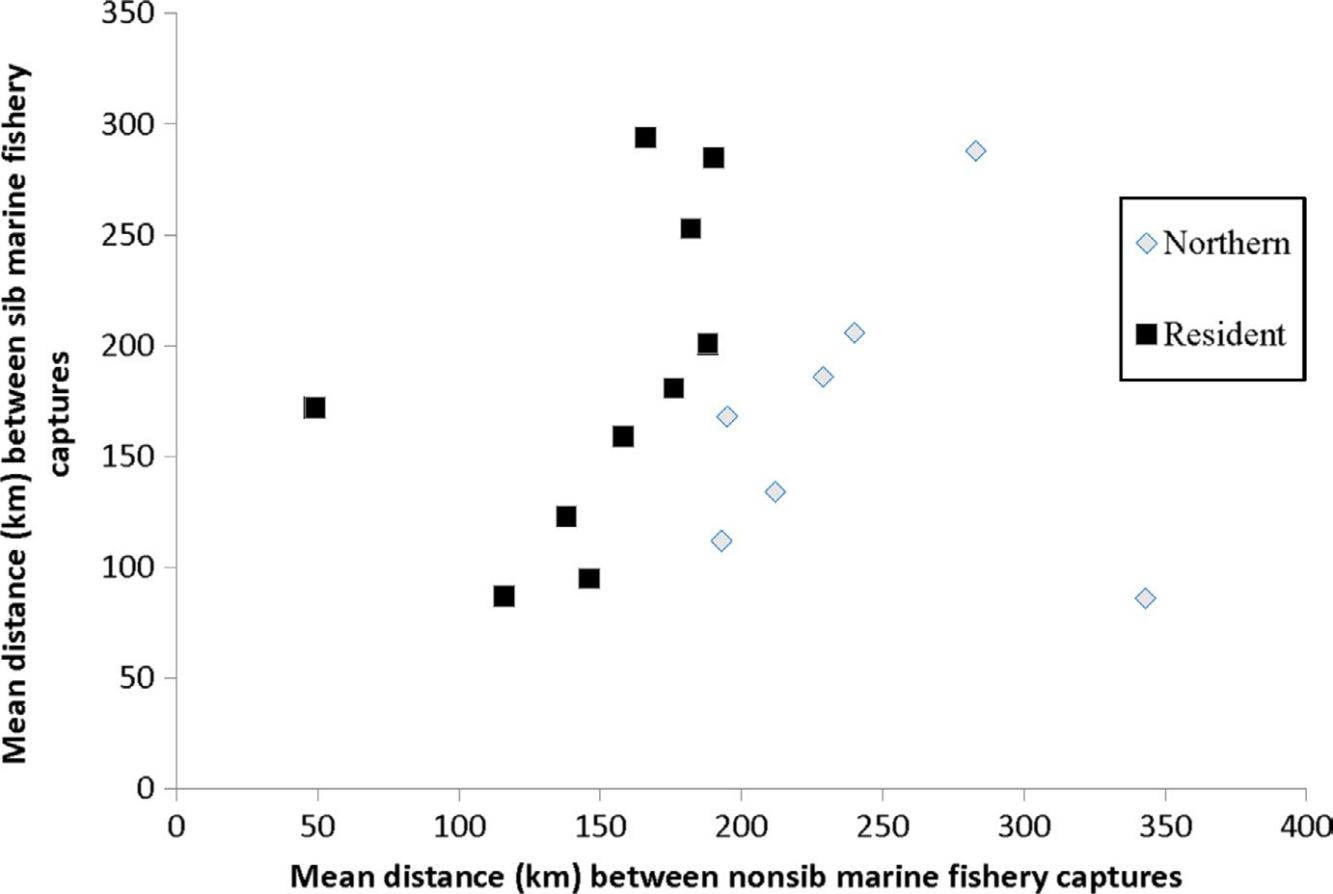
Comparison of mean distance between sib and nonsib 2017 marine fishery captures for 17 coho salmon populations in southern British Columbia

Northern migrating populations, those with higher proportions of fishery captures taking place in NC and CC fisheries, originated from Vancouver Island and the northern portion of the southern BC mainland. Resident populations, with fewer distant fishery recaptures, were located in the southern portion of the southern BC mainland, the lower Fraser River, and Boundary Bay, south of the mouth of the Fraser River (Table 2 and Figure 1). In northern migrating populations, the mean distance (mean ± standard error) between fishery captures of sibs (*n* = 458, mean = 166 ± 13 km) was significantly less than the mean distance between nonsibs (*n* = 41,208, mean = 199 ± 1 km; *t* = 2.47_(1, 41,664)_, *p* < .01; Table 2). This difference was the result of a disproportionate number of siblings being recovered jointly from relatively proximal northern or southern fishery locations, compared with nonsiblings which were more frequently sampled from more distal between‐region locations. For six of the seven northern migrating populations, the mean distance between sib captures was lower compared with nonsib capture distances, indicating a consistency of the association between migration pattern and relatedness for this set of populations (Figure 2).

**Table 2:** Number of progeny assigned to two parents in marine fishery samples by population (nt), total fishery recovery of progeny by broodstock male, number of pairwise nonsib fishery captures by population (*n*1), mean distance (km) between nonsib fishery capture, number of pairwise sib fishery captures by population (*n*2+), and mean distance (km) between sib fishery capture

Population	nt	Number of fishery captures per broodstock male										*n*1	Mean nonsibs	*n*2+	Mean sibs
		1	2	3	4	5	6	7	8	9	11				
Northern migrating															
Qualicum	81	48	12	3								3,219	229 (4)	21	186 (42)
Tenderfoot	37	22	6	1								657	212 (6)	9	134 (34)
Mamquam	17	7	6		1							127	240 (16)	9	206 (37)
Goldstream	12	8	2									64	343 (30)	2	86 (86)
Quinsam	102	66	10	4	1							5,123	193 (3)	28	112 (36)
Nitinat	24	16	4									272	283 (20)	4	288 (152)
Robertson	254	28	28	14	10	7	3	1	1	1	1	31,746	195 (2)	385	168 (15)
Robertson—female	254	28	20	15	21	4	5	1				31,804	195 (2)	327	186 (16)
Total—male only	527	195	68	22	12	7	3	1	1	1	1	41,208	199 (1)	458	166 (13)
Resident															
Norrish	40	15	6	3	1							759	146 (14)	21	95 (27)
Inch	43	22	3		2			1				867	188 (7)	36	201 (23)
Stave	18	14	2									151	190 (11)	2	285 (86)
Capilano	179	67	33	10	4							15,843	138 (1)	87	123 (13)
Serpentine	12	6	3									63	182 (17)	3	253 (127)
Salmon	6	4	1									14	49 (22)	1	172 (‐)
Coldwater	10	6	2									43	166 (21)	2	294 (77)
Conuma	14	7	2	1								86	116 (21)	5	87 (87)
Chilliwack	190	102	25	8	2		1					17,879	158 (1)	76	159 (16)
Chehalis	55	32	7	3								1,524	176 (4)	16	181 (42)
Total	574	280	85	25	9		1	1				37,229	151 (1)	249	150 (9)

Note

Standard error of the mean is in parentheses. No sibs were observed in fishery captures for the Puntledge River and Rosewall Creek populations, and all fishery captures of the Nicomekl River population occurred in the Juan de Fuca Strait fishery, so these populations were not included in the analysis. Northern migrating populations were defined as those populations where at least 5% of the observed fishery captures were observed in the North Coast and Central Coast fisheries.

In the resident populations, there was no significant difference in the mean distance between fishery captures of sibs (*n* = 249, mean = 150 ± 9 km) and nonsibs (*n* = 37,229, mean = 151 ± 1 km; *t* = 0.07_(1, 37,476)_, *p* > .10; Table 2). As expected, for the resident populations that reared primarily in southern BC marine waters, the overall intrapopulation means pairwise distance between fishery captures was lower than for the more migratory populations. Among the 10 resident populations, the mean distance between sib captures compared with nonsib capture distances was lower in three, approximately equivalent in three, and greater in four populations, indicating no apparent association between relatedness and migratory pattern within these populations (Figure 2).

### 3.2 Origin of 2017 hatchery broodstocks

For the 19 hatchery broodstocks sampled in 2017, 6,002 of 6,169 individuals were successfully genotyped (97.3% success rate). Four large hatchery broodstocks (Chilliwack River, Puntledge River, Capilano River, and Quinsam River) accounted for 49% of the total number of broodstock individuals sampled. Overall, approximately 75% of individuals genotyped (4,447 fish) were assigned to hatchery parents from 2014 and 2015. The assignment rate varied considerably among populations, ranging from 12.9% at Salmon River where the broodstock was obtained by seining in the river to 95.8% at Nitinat River where the broodstock swam into the hatchery facility (Table 3).

**Table 3:** Origin and age determined via PBT of individuals included in 2017 hatchery broodstocks

Population	Brood received	Brood genotyped	Assigned to	Number	% assigned	% not assigned
Nicomekl	72	72	2014 Nicomekl	19	26.4	72.2
			2014 Serpentine	1	1.4	
Serpentine	84	82	2014 Serpentine	17	20.7	79.3
Qualicum	556	525	2014 Qualicum	382	72.8	27.0
			2014 Rosewall	1	0.2	
Puntledge	796	758	2014 Puntledge	347	45.8	47.8
			2015 Puntledge	47	6.2	
			2014 Rosewall	1	0.1	
			2014 Robertson	1	0.1	
Quinsam	567	555	2014 Quinsam	469	84.5	11.5
			2015 Quinsam	22	4.0	
Rosewall	79	79	2014 Rosewall	69	87.3	10.2
			2015 Rosewall	2	2.5	
Capilano	744	739	2014 Capilano	590	79.8	19.5
			2015 Capilano	5	0.7	
Mamquam	48	47	2014 Mamquam	18	38.3	49.0
			2014 Tenderfoot	5	10.6	
			2014 Quinsam	1	2.1	
Tenderfoot	324	323	2014 Tenderfoot	251	77.7	22.0
			2014 Mamquam	1	0.3	
Nitinat	349	311	2014 Nitinat	287	92.3	4.2
			2015 Nitinat	10	3.2	
			2014 Puntledge	1	0.3	
Conuma	242	239	2014 Conuma	97	40.6	52.3
			2015 Conuma	17	7.1	
Robertson	228	228	2014 Robertson	189	82.9	17.1
Chehalis	467	466	2014 Chehalis	371	79.6	17.5
			2015 Chehalis	7	1.5	
			2014 Chilliwack	1	0.2	
			2014 Inch	2	0.4	
			2014 Norrish	1	0.2	
			2014 Stave	3	0.6	
Chilliwack	836	807	2014 Chilliwack	731	90.6	8.4
			2015 Chilliwack	6	0.7	
			2014 Chehalis	1	0.1	
			2014 Puntledge	1	0.1	
			2014 Qualicum	1	0.1	
Inch	208	208	2014 Inch	169	81.3	9.6
			2015 Inch	8	3.8	
			2014 Norrish	11	5.3	
Norrish	126	125	2014 Norrish	87	69.6	20.8
			2015 Norrish	2	1.6	
			2014 Inch	7	5.6	
			2014 Stave	1	0.8	
			2014 Qualicum	2	1.6	
Stave	154	152	2014 Stave	114	75.0	19.8
			2015 Stave	6	3.9	
			2014 Chehalis	2	1.3	
Coldwater	227	224	2014 Coldwater	55	24.6	75.4
Salmon	62	62	2014 Salmon	8	12.9	87.1

Note

Percentage not assigned is the percentage of the 2017 hatchery broodstock fish that could not be assigned to any hatchery broodstock genotyped in either 2014 or 2015.

Hatchery‐origin jacks or jills comprised an average 2.2% of broodstock fish, with the highest value (6.2%) observed for Puntledge River (Table 3). Hatchery strays from sampled populations were incorporated into broodstocks at an average rate of 1.0% (45 strays in 4,447 individuals), with 40% of all straying occurring between Inch Creek and Norrish Creek. Higher rates of straying were observed among Lower Fraser populations compared with other regions.

### 3.3 Population and parental male productivity

A total of 6,943 progeny from 2014 hatchery parents were genotyped from escapement samples in 2016 (9.0% of progeny) and 2017 (70.5%), and fishery samples in 2017 (20.5%; Table 4). There was inter‐ and intrapopulation variation in age of maturity, the relative contributions to fisheries versus escapement, and productivity of individual male spawners. Populations with large broodstocks naturally produced more progeny (Tables 3 and 4), but number of progeny produced by individual male spawner provided a standardized measure of productivity.

**Table 4:** Fishery and escapement distribution of progeny recovery for the 2014 coho salmon hatchery broodstocks

Population	*n*	Total PBT	Total 2017 fishery PBT	Total jack 2016 escapement PBT	Total 2017 escapement PBT	Total escapement PBT
Robertson	120	607	270	66	271	337
Conuma	24	111	13	0	98	98
Nitinat	291	309	24	0	285	285
Qualicum	224	626	85	80	461	541
Quinsam	269	838	118	68	652	720
Puntledge	372	390	4	20	366	386
Rosewall	60	78	7	0	71	71
Goldstream	98	29	12	17	0^a^	17
Mamquam	36	36	17	0	19	19
Tenderfoot	126	307	48	13	246	259
Capilano	338	804	199	84	521	605
Nicomekl	28	24	7	0	17	17
Serpentine	40	30	12	0	18	18
Chilliwack	443	1,367	304	171	892	1,063
Inch	84	430	112	66	252	318
Norrish	63	171	83	1	87	88
Stave	53	253	22	19	212	231
Chehalis	189	457	71	17	369	386
Salmon	31	14	6	0	8	8
Coldwater	40	62	10	0	52	52
Total	2,929	6,943	1,424	622	4,897	5,519

Note

*n* is number of genotyped males in 2014 hatchery broodstocks, and total PBT is the total number of progeny identified for all genotyped males of a broodstock. Progeny numbers recovered from 2017 fishery sampling and 2016 and 2017 escapement sampling of broodstock and nonbroodstock fish are also provided. The hatchery broodstock was considered part of the escapement.

a Goldstream River broodstock was not surveyed or genotyped in 2017.

The Chilliwack River population had the highest proportion of age‐2 progeny in 2016 escapement sampling (12.5% of 1,367 individuals), none were identified in several populations (Table 4). The Robertson Creek population made the highest relative contribution to 2017 fisheries versus escapement (44.5% of 607 fish), whereas the lowest relative fishery contribution was from Puntledge River (1.0% of 390 fish; Table 4). In Robertson Creek, the average male spawner produced 2.25 fish recovered from fisheries, 4.3 times the average male contribution in the other 19 populations, with most of the fish sampled in marine fisheries (Table 5). The Inch Creek and Norrish Creek broodstocks also displayed substantial contributions per male spawner to fisheries (1.33 and 1.32 individuals, respectively), predominantly in freshwater locations (0.92 and 0.88 individuals, respectively). With respect to the 2017 escapements, the Conuma River and Stave River broodstock males made the highest relative contributions (4.08 and 4.00 individuals, respectively). For 2017 fishery and escapement samples combined, male productivity was highest for the Inch Creek and Robertson Creek populations (5.12 and 5.06, respectively; Table 5).

**Table 5:** Progeny produced per spawning genotyped male (R/S) in 2014 broodstocks by recovery location including 2017 fisheries and 2016 and 2017 escapements comprising both broodstock and nonbroodstock fish for 20 coho salmon populations

Population	2017 Fisheries			Hatchery broodstocks and nonbroodstock escapements			Total
	Marine	Fresh	All	Jacks	Adults	All	
Robertson	2.18	0.07	2.25	0.55	2.26	2.81	5.06
Conuma	0.54	0.00	0.54	0.00	4.08	4.08	4.62
Nitinat	0.08	0.00	0.08	0.00	0.98	0.98	1.06
Qualicum	0.37	0.01	0.38	0.36	2.06	2.42	2.80
Quinsam	0.39	0.05	0.44	0.25	2.42	2.67	3.11
Puntledge	0.01	0.00	0.01	0.05	0.99	1.04	1.05
Rosewall	0.12	0.00	0.12	0.00	1.18	1.18	1.30
Goldstream^a^	0.12	0.00	0.12	0.17	0.00^a^	0.17	0.29
Mamquam	0.47	0.00	0.47	0.00	0.53	0.53	1.00
Tenderfoot	0.30	0.08	0.38	0.10	1.95	2.05	2.43
Capilano	0.58	0.01	0.59	0.25	1.54	1.79	2.38
Nicomekl	0.25	0.00	0.25	0.00	0.61	0.61	0.86
Serpentine	0.30	0.00	0.30	0.00	0.45	0.45	0.75
Chilliwack	0.42	0.27	0.69	0.39	2.01	2.40	3.09
Inch	0.41	0.92	1.33	0.79	3.00	3.79	5.12
Norrish	0.44	0.88	1.32	0.02	1.35	1.37	2.56
Stave	0.16	0.26	0.42	0.36	4.00	4.36	4.78
Chehalis	0.30	0.08	0.38	0.09	1.95	2.04	2.42
Salmon	0.19	0.00	0.19	0.00	0.26	0.26	0.45
Coldwater	0.25	0.00	0.25	0.00	1.30	1.30	1.55
Mean	0.39	0.13	0.52	0.17	1.65	1.82	2.34

a 2017 Goldstream River broodstock was not sampled or genotyped.

Extensive intrapopulation variation was also observed for productivity traits. A total of 42 males over nine populations produced significantly more jack individuals than did the average male in the population (Table S1). One Chilliwack River male spawner produced 17 total progeny among all samples, of which 12 were jacks. The relative fishery and escapement contributions also varied, with 26 males from 11 populations producing significantly more fishery recoveries than expected (Table S2). For example, of 78 males in the Robertson Creek broodstock that produced four or more sampled progeny, one had 12 progeny recovered from fisheries, but only two progeny from escapement sampling (Table S2). In comparison, the average Robertson Creek male contributed 2.25 and 2.81 progeny to fishery and escapement samples, respectively (Table 5). An additional five Robertson Creek males made disproportionately high contributions to fishery samples.

Just as some males made disproportionate contributions to fishery captures, so too did others to escapements. Six males over four populations contributed significantly more escapement progeny than would be expected (Table S3). Some males contributed 14–21 total progeny to escapement samples, but none to fishery samples.

### 3.4 Effect of migration return and spawn time on progeny number and timing

The average number of progeny recovered from 2014 Capilano broodstock differed among the three parental male return time groups (Table 6). Early males produced significantly more total progeny than late males (*F*_(1, 202)_ = 13.3, *p* < .01), as did mid males (*F*_(1, 301)_ = 11.2, *p* < .01). Most progeny were recovered in the 2017 escapement samples, with 50% more progeny produced by each early male than mid male (*F*_(1, 167)_ = 4.22, *p* < .05). Significant differences also existed between mid and late males (*F*_(1, 301)_ = 24.55, *p* < .001), and early males contributed three times as many progeny as late males (*p* < .01; Table 6). Thus, the small number of early return males spawned in 2014 made a disproportionately large contribution to the 2017 escapement, making the temporal distribution of progeny across the escapement and also to 2017 fisheries of interest. In the escapement, the majority of progeny were recovered from within the paternal return time (Table 7), indicating an influence of paternal return time on progeny return time.

**Table 6:** Time of return or spawning for progeny of early‐, mid‐, and late‐returning Capilano River and October‐, November‐, and December‐spawning Chilliwack River males of the 2014 broodstock

	Capilano			Chilliwack		
	Early	Mid	Late	October	November	December
*n*	35	134	169	75	160	208
PBT/S jacks (esc)	0.06	0.30	0.25	0.73	0.40	0.25
PBT/S adults (esc)	3.00	1.96	0.91	3.45	1.96	1.72
PBT/S escapement	3.06	2.26	1.16	4.18	2.36	1.97
PBT/S fisheries	0.29	0.51	0.71	0.63	0.78	0.64
PBT/S Total	3.35	2.77	1.87	4.81	3.14	2.61

Note

Number of parental males in each return/spawn time is given (*n*), and number of progeny recovered per male (PBT/S) is shown for the 2016 escapement samples (jacks), the 2017 escapement samples (adults), total escapement samples, the 2017 fishery samples, and all samples combined.

**Table 7:** Number (*n*) and percentage of progeny among return time windows for Early, Mid, and Late 2014 Capilano River parents recovered in escapement (2016 and 2017) and fishery samples (2017)

Source	Timing	2014 Capilano		
		Early	Mid	Late
2016 escapement	*n*	2	41	41
	Early	0.0	0.0	0.0
	Middle	50.0	85.4	12.2
	Late	50.0	14.6	87.8
2017 fisheries	*n*	10	69	120
	May	0.0	0.0	0.8
	June	0.0	17.4	4.2
	July	20.0	31.9	20.8
	August	70.0	24.6	32.5
	September	0.0	23.2	31.7
	October	10.0	1.4	10.0
	November	0.0	1.4	0.0
2017 escapement	*n*	105	255	151
	Early	54.3	25.1	0.0
	Middle	40.0	62.0	3.3
	Late	5.7	12.9	96.7

In contrast with contributions to the escapement, fishery recoveries per male increased significantly with return date for the three return groups (*F*_(2, 335)_ = 3.86, *p* < .03), with early males contributing an average 0.29 progeny and late males 0.71 progeny (*F*_(1, 202)_ = 5.99, *p* < .02). Capilano River progeny were recovered in fishery catches from May through November 2017, with a significant difference in monthly distribution observed among the 2014 paternal groups ($\chi_{\left(12\right)}^{2}$ = 29.1, *p* < .01). The temporal differences in capture were significant between both the early and mid return groups ($\chi_{\left(5\right)}^{2}$ = 13.0, *p* < .03) and the mid and late groups ($\chi_{\left(6\right)}^{2}$ = 19.7, *p* < .01). Progeny from the early males were concentrated in July and August harvest, whereas progeny of mid males were mainly harvested from June through September (Table 7). As might be expected, progeny of late males were harvested disproportionately in later months (July through October; Table 7). Thus, the temporal distribution of progeny fishery captures was related to paternal return time. The spatial distribution of progeny groups among fisheries did not vary significantly, although there was an indication that progeny from early males were disproportionately caught in the JDF fishery.

**Figure 3:**
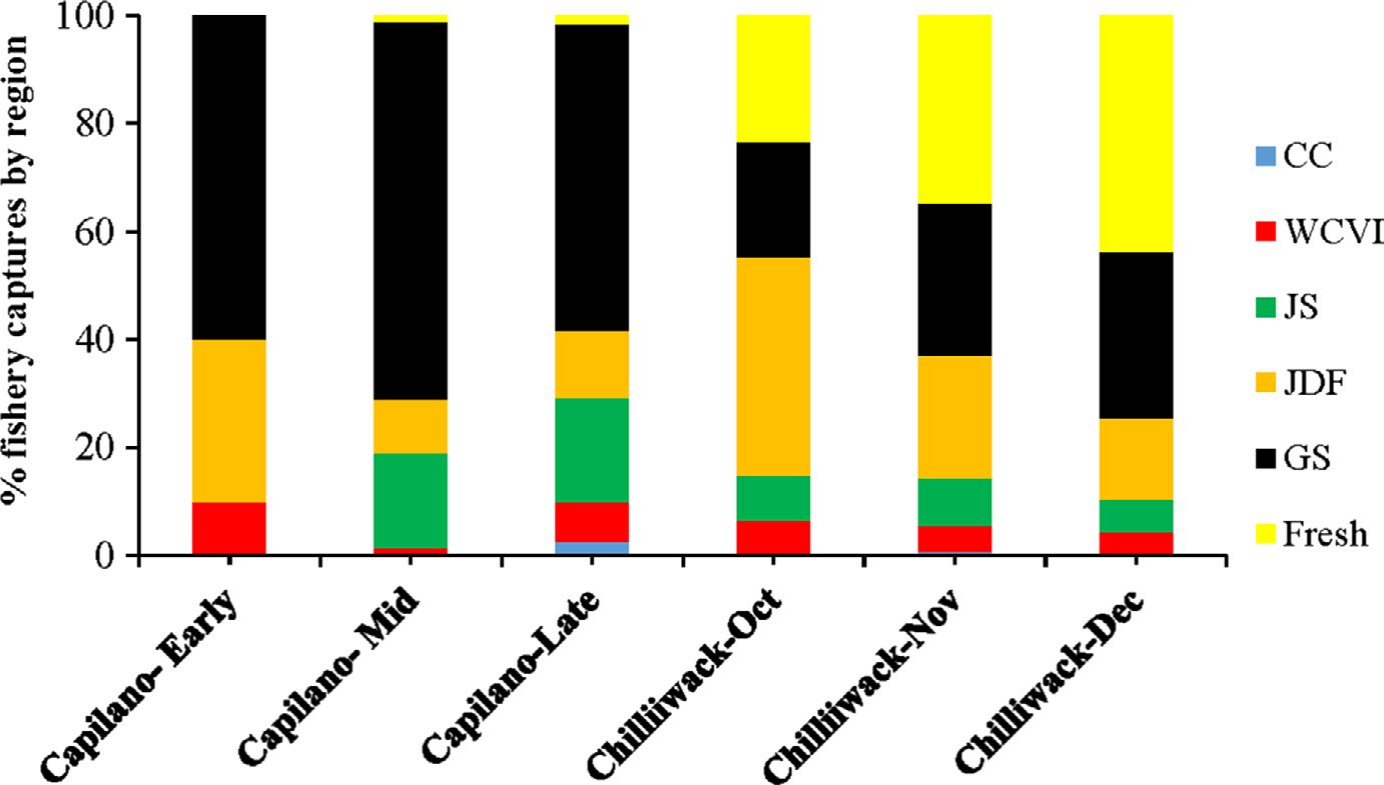
Percentage distribution of PBT fishery recoveries of offspring originating from early‐, mid‐, and late‐returning Capilano River and October‐, November‐, and December‐spawning Chilliwack River genotyped males from the 2014 hatchery broodstocks

At the Chilliwack River hatchery, the 2014 broodstock was spawned from October through December, and monthly spawn groups were defined because migration return time had not been monitored. There was no evidence for a difference in progeny distribution in 2017 fisheries between November and December spawners ($\chi_{\left(5\right)}^{2}$ = 5.2, *p* > .10), but progeny of October spawners were observed more frequently in the JDF fishery and less frequently in freshwater fisheries than those of the later spawn groups ($\chi_{\left(5\right)}^{2}$ = 12.7, *p* < .03; Figure 3). Progeny were recovered in fishery catches from June through November 2017, with a significant difference in monthly progeny composition in the catch ($\chi_{\left(10\right)}^{2}$ = 50.5, *p* < .001). Monthly catch distributions of progeny differed significantly between October and November spawners ($\chi_{\left(4\right)}^{2}$ = 23.2, *p* < .001) and between November and December spawners ($\chi_{\left(5\right)}^{2}$ = 19.6, *p* < .001). Progeny from October spawners were concentrated in the September harvest, whereas progeny from November spawners were predominantly caught in October. Thus, progeny harvest time shifted progressively later with paternal spawn time.

Similar to the Capilano River population, the total number of progeny produced per Chilliwack River male was significantly greater in October than November (*F*_(1, 233)_ = 18.52, *p* < .001), and greater in November than December (*F*_(1, 365)_ = 8.14, *p* < .01; Table 6). Progeny recoveries per male in fishery samples were similar among the three spawning groups. In the escapement, both jack and adult contributions decreased over time among paternal spawn times (Table 6). November spawners contributed fewer adult fish per male to the 2017 escapement than did October spawners (*F*_(1, 233)_ = 27.30, *p* < .001), and December spawners contributed fewer than November spawners (1.97, 208 males; *F*_(1, 365)_ = 6.64, *p* < .02).

## 4 DISCUSSION

This study illustrated that significant inter‐ and intrapopulation variability in coho salmon life history characteristics was associated with differences in overall contributions to and patterns of harvest. Within populations, productivity and life history traits differed among paternal families, suggesting that much of the variation may have a genetic basis and therefore be subject to change not only by hatchery broodstock management but also by harvest pressures. However, it is important to note that the standard mating design employed for these coho populations was single‐pair crosses, with each male and female brood parent mainly having only one mate. Thus, the “paternal” effects noted in this study actually embodied both additive and nonadditive genetic effects as well the influence of the common incubation and rearing environment experienced by each family.

In general, the large hatchery populations of this study have been managed primarily for harvest goals, whereas some populations with small broodstocks were initiated for conservation and/or assessment purposes. The proportion of hatchery‐origin fish in the 2017 broodstocks reflected this dichotomy in purpose, with large populations generally dominated (>70%) by hatchery fish and only a few populations that contained >50% natural‐origin fish.

In locations where the broodstock was seined from the local river (Salmon River, Eagle River, Serpentine River, Nicomekl River), hatchery‐origin fish were <30%, whereas in those locations where the broodstock swam into the hatchery facility (Robertson Creek, Inch Creek, Nitinat River), hatchery‐origin fish exceeded 80%. With both hatchery‐ and natural‐origin fish contributing to spawning in the natural and hatchery environments in these populations, little genetic differentiation between fish originating from the two environments would be expected, although the degree of domestication for the entire river population may be high for the large enhancement programs. A genome‐wide lack of genetic differentiation based on spawning origin was confirmed for two populations included in this study (Quinsam River, Capilano River), although parallel epigenetic differences between hatchery‐ and natural‐origin origin fish of the two populations were noted (Le Luyer et al., 2017).

Strays were incorporated into the 2017 hatchery broodstocks at a rate of 1.0%, similar to the rate observed in the 2017 nonbroodstock escapement (0.7%, 10 strays identified in 1,530 assigned individuals) in many of the same populations (Beacham et al., 2019). Labelle (1992) reported that straying in three populations on the southeast coast of Vancouver Island was <2%, similar to the rate observed in the current study. The highest stray rates were observed between the geographically proximate Inch Creek and Norrish Creek populations, with both creeks draining into Nicomen Slough <1 km apart. On average, strays were observed to be a minor component (<1%) of the populations surveyed in the study.

Nominally, poor productivity of the 2014 broodstocks was observed at the Nitinat River and Puntledge River hatcheries. The apparent poor productivity of the Nitinat River hatchery was accounted for by the culling of approximately 70% of the 1.2 million eggs taken due to high levels of bacterial kidney disease infection rates in female parents, the highest rate observed in any of the populations screened (P. Ackerman, Fisheries and Oceans Canada, pers. comm.). The number of eggs taken in 2017 was substantially reduced in order to reduce the volume of discarded eggs. Water temperatures at the Puntledge River hatchery become too high during the summer for juvenile rearing, so approximately 85% of the production from the 2014 broodstock was released as fry, likely leading to lower freshwater survival and productivity for this population. Subsequently, an alternative rearing strategy has been developed whereby some juveniles from this population are transferred to an alternative site for summer rearing, and transferred back to the Puntledge River hatchery prior to release.

Variation in migration distance, as evidenced by fishery recaptures, occurred among and within populations. Populations making substantial fishery contributions standardized to broodstock size included both northern migrating and resident populations, with Robertson Creek making very substantial contributions in a diverse array of fishing locations. For the northern migrating populations, in which fish could be recovered in distal northern harvest sites as well as southern locations proximal to natal streams, there was a greater tendency for siblings than nonsiblings to be captured in the same region. The sibs did not have to school in close proximity to each other, only reside in the same fishing area at time of capture. Sibling recognition in coho salmon has previously been reported (Quinn & Busack, 1985; Quinn & Hara, 1986), as has sibling recognition in other salmonids (Olsén, 1989). However, similar migratory tendencies in siblings may simply reflect phenotypic and or genetic similarities rather than kin recognition (Dodson, Aubin‐Horth, Thćriault, & Paez, 2013; Fraser, Duchesne, & Bernatchez, 2005).

Juvenile body size may have an important effect on migratory phenotype (Dodson et al., 2013). Beacham et al. (2016) reported that there was a relationship between timing of northward migration and juvenile body size in coho salmon, with larger individuals migrating northward earlier than smaller individuals from the same populations. It may be that long‐distance migration in coho salmon is mediated via growth rate, with faster‐growing individuals having a greater propensity for longer northward migration. Growth rate in salmonids has a heritable component (Gutierrez, Yáňez, Fukui, Swift, & Davidson, 2015; Nilsson, 1990), and the smaller distance in fishery captures among sibs may reflect similarity in growth rate and body size. In resident coho populations, growth rates may have been insufficient to trigger long‐distance migration in even the fastest growing members, or the migration routes undertaken did not lead to capture in northern fisheries.

Return time and spawning time in coho salmon populations also have a genetic basis (Ford et al., 2006). In a population in Oregon with at least a five‐month interval in time of return and with three defined return time groups, Tipping and Busack (2004) reported that 57% of 3‐year‐old adult fish returned in the return time window of origin and that many of the early‐ and late‐origin fish returned in the middle time period (mid‐October through November). In our study, for two populations with an extended in return time or spawning time, paternal time group influenced both the total number and timing of progeny recovered. Early‐returning Capilano and early‐spawning Chilliwack males were fewer in number but produced more progeny per capita than later males. For Capilano, progeny tended to return in the paternal time window, but there was significant overlap in return time of progeny from the early and mid‐groups. In contrast, only 3% of late‐origin offspring returned at an earlier time.

In contrast to total contributions from Capilano males, fishery contributions increased over time. Thus, from a viewpoint of maximizing hatchery contributions to fisheries and minimizing escapement to the natural environment, later spawning of the Capilano population may be preferable. Whereas the selective pressure of fishing on later spawning fish might favor an earlier return time, only a small proportion of the broodstock was early migrating. This may reflect a relatively low fitness of early fish when they spawn in the natural environment. Early‐returning Capilano River coho salmon are smaller than later returning fish in the population, and smaller than the typical body size in other local coho salmon populations (A. Uittenbogaard, Fisheries and Oceans Canada, pers. comm.). Since small body size in salmonids is a trait often related to low fitness in the natural spawning environment, the small body size may not hinder hatchery reproductive success but be selected against in the natural environment.

At the Chilliwack River, a modest fidelity to 2014 parental spawning month was observed in the 2017 progeny spawners, with an average of 62% of spawning in the same month as their parents. Higher progeny numbers per early‐spawning male were the result of their contributions to 2017 escapement across all spawn time windows. In contrast to the Capilano population, parental contributions to fisheries did not increase over time. If this is a typical result, temporally selective harvest is unlikely to exert a strong pressure for early spawning in this population. The October 2014 males were the most productive on an individual basis but constituted only 17% of the broodstock and did not contribute disproportionately to the escapement. The current hatchery practice of apportioning broodstock relative to monthly abundance would seem to be a prudent course to follow to maintain stability in timing of return and overall genetic diversity within the population.

Chilliwack River males that spawned in October also produced more jacks than did later spawning males. Heritability of jacking has been documented in salmonids (Berejikian et al., 2010; Heath, Rankin, Bryden, Heath, & Shrimpton, 2002; Iwamoto, Alexander, & Hershberger, 1984), so it was expected that some males should contribute disproportionately to jack returns. In coho salmon, families that produce some jack progeny do not necessarily produce fewer adult fish than families without jack returns and the inclusion of jacks in hatchery broodstocks may be important in maintaining effective population size (Van Doornik, Ford, & Teel, 2002).

Within some populations, some males contributed disproportionately to either fishery or escapement samples, indicating that fishery capture may have a heritable basis and/or be affected by early common environmental effects. Vulnerability to angling and net fisheries may be heritable and may be related to behavioral aggressiveness and high growth rates (Biro & Post, 2008; Cooke, Suski, Ostrand, Wahl, & Philipp, 2007). Fishery harvest is generally managed to exploit larger rather than smaller individuals in the populations, and an intrapopulation relationship between large size and increased catchability may increase selection intensity for smaller size within a population. With efficient fishery capture and the desired outcome for hatchery populations reared for harvest augmentation, the retention of the genetic basis for increased catchability is of importance. In theory, analysis of fishery samples could be used to identify families that contribute disproportionately to fisheries and enable the subsequent selection of corresponding family members for broodstock use. On a practical basis, the use of PBT on potential brood fish to identify individuals from families with low representation in the escapement may be sufficient to maintain the genes for increased capture likelihood in a hatchery population. Ultimately, determination of the genomic basis for catchability may enable marker‐assisted or genomic selection programs.

The comprehensive evaluation of hatchery coho salmon populations to fishery contributions in this study is valuable for hatchery management purposes because few Canadian coho salmon populations are tagged with coded‐wire tags, and current tag recovery rates are low (Beacham et al., 2019). The great variability in productivity observed among and within hatchery populations reflects the adult fishery and escapement returns from a single spawning cohort. If ongoing analysis confirms that the differential contributions to harvest are stable characteristics, hatchery programs may be modified to support specific harvest objectives. For example, increased production from the Capilano River, Chilliwack River, Inch Creek, and Norrish Creek populations would support increased harvest in the SOG recreational fishery. Spawners in each of these four broodstocks contributed relatively high numbers (0.41–0.58) of progeny to marine fisheries, with significant loading in the SOG fishery (39%–63% of total identified hatchery contributions). In fact, the Capilano and Chilliwack populations accounted for approximately 60% (214 of 363) of all hatchery fish identified in the SOG fishery.

Broodstock selection within hatchery populations might also be applied to amplify fishery contributions and reduce escapement numbers. The detrimental effects of hatchery‐origin spawners in the natural spawning environment can be mitigated by restricting their numbers on the spawning grounds, an objective at least partially met by increased catchability of hatchery fish. This study provided evidence of low stray rates among the hatchery populations themselves, but rates of straying into natural populations in close proximity to the hatcheries were not measured. Even low rates of straying from highly successful and abundant hatchery populations into small natural populations can have important genetic impact (Keefer & Caudill, 2014). The evidence for family variation in coho salmon catchability from this study indicated that further examination of this trait is merited. Fishery selection will tend to favor lowered catchability over time in hatchery populations. To the extent that catchability may be associated with other beneficial traits such as growth rate and body size, maintenance of high catchability in hatchery populations may be a useful objective not only for maximizing harvest benefits but also for maintaining population viability.

Migratory distance and route also affected coho salmon fishery contributions and might be influenced by broodstock selection, particularly within the northern migrating populations. Timing of adult migration and spawning could be manipulated within populations to increase fishery contributions or to counteract temporally selective fishing patterns. In the Capilano River population, the spawning of relatively more late return fish could lead to increased harvest contributions and lower escapement levels.

The strong genetic basis for, and existence of both genetic and phenotypic correlations among, life history traits in salmonids makes the outcome of hatchery broodstock‐selective efforts unpredictable, especially when combined with selective fishery forces that may be poorly characterized (Tillotson & Quinn, 2018). Broodstock manipulation within hatchery populations should be approached with caution and on an experimental basis until greater understanding of consequences is gained. Nevertheless, inadvertent selection in both hatchery production and the harvest of salmon is generally viewed as a force that reduces genetic diversity within and among Pacific salmon populations (Moore, McClure, Rogers, & Schindler, 2010). The use of parentage‐based genetic analysis increases our ability to identify, monitor, and possibly maintain variation within populations as they face the future challenges of environmental degradation, climate change, and ongoing harvest and predation.

## CONFLICT OF INTEREST

None declared.

## AUTHORS' CONTRIBUTIONS

T.D.B. and R.E.W. designed the project and wrote the manuscript. D.W. and C.L. led data collection. K.J. and B.M. led genotyping acquisition. C.W and J.R.C. led data processing and analysis.

## Data Availability

Multilocus genotypes for all sampled jacks, as well as individuals in fishery and escapement samples, are available at DRYAD https://doi.org/10.5061/dryad.6c31bf2 Data files: 2016_Coho_Jacks_rubias. 2016‐17_Coho_Fishery_Samples_rubias, 2017_Coho_Escapement_rubias. Multilocus genotypes for 2017 hatchery broodstocks are deposited in the Data package title: Data from: Variation in migration pattern, broodstock origin, and family productivity of coho salmon hatchery populations in British Columbia, Canada, derived from parentage‐based tagging. Journal: Ecology and Evolution Provisional https://doi.org/10.5061/dryad.3g1r4v3 Data files: 2017 Coho PBT Baseline.

## References

[ece35530-bib-0001] Anderson, E. C. , & Garza, J. C. (2006). The power of single‐nucleotide polymorphisms for large‐scale parentage inference. Genetics, 172(4), 2567–2582. 10.1534/genetics.105.048074 16387880 PMC1456362

[ece35530-bib-0002] Beacham, T. D. , Beamish, R. J. , Neville, C. M. , Candy, J. R. , Wallace, C. , Tucker, S. , & Trudel, M. (2016). Stock‐specific size and migration of juvenile coho salmon in British Columbia and southeastern Alaskan waters. Marine and Coastal Fisheries: Dynamics, Management, and Ecosystem Science, 8, 292–314.

[ece35530-bib-0003] Beacham, T. D. , Wallace, C. , Jonsen, K. , McIntosh, B. , Candy, J. R. , Willis, D. , … Withler, R. E. (2019). Comparison of coded‐wire tagging with parentage‐based tagging and genetic stock identification in a large‐scale coho salmon fisheries application in British Columbia, Canada. Evolutionary Applications, 12, 230–254. 10.1111/eva.12711 30697336 PMC6346672

[ece35530-bib-0004] Beacham, T. D. , Wallace, C. , MacConnachie, C. , Jonsen, K. , McIntosh, B. , Candy, J. R. , … Withler, R. E. (2017). Population and individual identification of Coho salmon in BC through parentage‐based tagging and genetic stock identification: An alternative to coded‐wire tags. Canadian Journal of Fisheries and Aquatic Sciences, 74, 1391–1410.

[ece35530-bib-0005] Beacham, T. D. , Wallace, C. , MacConnachie, C. , Jonsen, K. , McIntosh, B. , Candy, J. R. , & Withler, R. E. (2018). Population and individual identification of Chinook Salmon in British Columbia through parentage‐based tagging and genetic stock identification with single nucleotide polymorphisms. Canadian Journal of Fisheries and Aquatic Sciences, 75, 1096–1105. 10.1139/cjfas-2017-0168

[ece35530-bib-0006] Berejikian, B. A. , Endicott, D. M. , Van Doornik, R. C. , Hoffnagle, T. L. , Tezak, E. P. , Moore, M. E. , & Atkins, J. (2010). Mating success of alternative male phenotypes and evidence for frequency‐dependent selection in Chinook salmon, *Oncorhynchus tshawytscha* . Canadian Journal of Fisheries and Aquatic Sciences, 67, 1933–1941. 10.1139/F10-112

[ece35530-bib-0007] Biro, P. A. , & Post, J. R. (2008). Rapid depletion of genotypes with fast growth and bold personality traits from harvested fish populations. Proceedings of the National Academy of Sciences of the United States of America, 105, 2919–2922. 10.1073/pnas.0708159105 18299567 PMC2268560

[ece35530-bib-0008] Cooke, S. J. , Suski, C. D. , Ostrand, K. G. , Wahl, D. H. , & Philipp, D. P. (2007). Physiological and behavioral consequences of long‐term artificial selection for vulnerability to recreational angling in a teleost fish. Physiological and Biochemical Zoology, 80, 480–490. 10.1086/520618 17717811

[ece35530-bib-0009] Davison, R. J. , & Sattherwaite, W. H. (2017). Life history effects on hatchery contributions to ocean harvest and natural‐area spawning. Canadian Journal of Fisheries and Aquatic Sciences, 74, 1575–1587. 10.1139/cjfas-2016-0457

[ece35530-bib-0011] Dodson, J. J. , Aubin‐Horth, N. , Thćriault, V. , & Paez, D. J. (2013). The evolutionary ecology of alternative migratory tactics in salmonid fishes. Biological Reviews, 88, 602–625. 10.1111/brv.12019 23347290

[ece35530-bib-0012] Fisher, J. , Trudel, M. , Ammann, A. , Orsi, J. A. , Piccolo, J. , Bucher, C. , … Welch, D. W. (2007). Comparison of the coastal distributions and abundances of juvenile Pacific salmon from Central California to the Northern Gulf of Alaska In Grimes C. B., Brodeur R. D., Haldorson L. J., & McKinnell S. M. (Eds.), The ecology of juvenile salmon in northeast Pacific Ocean: Regional comparisons. American Fisheries Society Symposium (Vol. 57, pp. 31–80). Bethesda, MD: American Fisheries Society.

[ece35530-bib-0013] Fisher, R. A. (1954). Statistical methods for research workers. London, UK: Oliver and Boyd.

[ece35530-bib-0014] Ford, M. J. , Fuss, H. , Boelts, B. , LaHood, E. , Hard, J. , & Miller, J. (2006). Changes in run timing and natural smolt production in a naturally spawning coho salmon (*Oncorhynchus kisutch*) population after 60 years of intensive hatchery supplementation. Canadian Journal of Fisheries and Aquatic Sciences, 63, 2343–2355.

[ece35530-bib-0016] Fraser, D. J. , Duchesne, P. , & Bernatchez, L. (2005). Migratory charr schools exhibit population and kin associations beyond juvenile stages. Molecular Ecology, 14, 3133–3146. 10.1111/j.1365-294X.2005.02657.x 16101779

[ece35530-bib-0017] Gutierrez, A. P. , Yáňez, J. M. , Fukui, S. , Swift, B. , & Davidson, W. S. (2015). Genome‐wide association study (GWAS) for growth rate and age at sexual maturation in Atlantic salmon (*Salmo salar*). PLoS ONE, 10(3), e0119730 10.1371/journal.pone.0119730 25757012 PMC4355585

[ece35530-bib-0018] Hard, J. J. , Gross, M. R. , Heino, M. , Hilborn, R. , Kope, R. G. , Law, R. , & Reynolds, J. D. (2008). Evolutionary consequences of fishing and their implications for salmon. Evolutionary Applications, 1, 388–408. 25567639 10.1111/j.1752-4571.2008.00020.x PMC3352430

[ece35530-bib-0019] Hartt, A. C. , & Dell, M. B. (1986). Early oceanic migrations and growth of juvenile Pacific salmon and steelhead trout. Bulletin of the International North Pacific Fisheries Commission, 46, 1–105.

[ece35530-bib-0020] Heath, D. D. , Rankin, L. , Bryden, C. , Heath, J. , & Shrimpton, J. (2002). Heritability and Y‐chromosome influence in the jack male life history of Chinook salmon (*Oncorhynchus tshawytscha*). Heredity, 89, 311–317. 10.1038/sj.hdy.6800141 12242648

[ece35530-bib-0021] Hess, J. E. , Ackerman, M. W. , Fryer, J. K. , Hasselman, D. J. , Steele, C. A. , Stephenson, J. J. , … Narum, S. R. (2016). Differential adult migration‐timing and stock‐specific abundance of steelhead in mixed stock assemblages. ICES Journal of Marine Science, 73, 2606–2615. 10.1093/icesjms/fsw138

[ece35530-bib-0022] Iwamoto, R. N. , Alexander, B. A. , & Hershberger, W. K. (1984). Genotypic and environmental effects on the incidence of sexual precocity in coho salmon *(Oncorhynchus kisutch)* . Aquaculture, 43, 105–121. 10.1016/0044-8486(84)90015-2

[ece35530-bib-0023] Jones, O. W. , & Wang, J. (2010). COLONY: A program for parentage and sibship inference from multilocus genotype data. Molecular Ecology Research, 10, 551–555. 10.1111/j.1755-0998.2009.02787.x 21565056

[ece35530-bib-0024] Keefer, M. L. , & Caudill, C. C. (2014). Homing and straying by anadromous salmonids: A review of mechanisms and rates. Reviews in Fish Biology and Fisheries, 24, 333–368. 10.1007/s11160-013-9334-6

[ece35530-bib-0025] Kendall, N. W. , & Quinn, T. P. (2009). Effects of population‐specific variation in age and length on fishery selection and exploitation rates of sockeye salmon (*Oncorhynchus nerka*). Canadian Journal of Fisheries and Aquatic Sciences, 66, 896–908.

[ece35530-bib-0026] Kendall, N. W. , & Quinn, T. P. (2011). Length and age trends of Chinook Salmon in the Nushagak River, Alaska, related to commercial and recreational fishery selection and exploitation. Transactions of the American Fisheries Society, 140, 611–622. 10.1080/00028487.2011.585575

[ece35530-bib-0027] Labelle, M. (1992). Straying patterns of coho salmon (*Oncorhynchus kisutch*) stocks from southeast Vancouver Island, British Columbia. Canadian Journal of Fisheries and Aquatic Sciences, 49, 1843–1855.

[ece35530-bib-0028] Le Luyer, J. , Laporte, M. , Beacham, T. D. , Kaukinen, K. H. , Withler, R. E. , Koop, B. , & Bernatchez, L. (2017). Parallel epigenetic modifications induced by hatchery rearing in a Pacific salmon. Proceedings of the National Academy of Sciences of the United States of America, 114, 12964–12969. 10.1073/pnas.1711229114 29162695 PMC5724268

[ece35530-bib-0029] Moore, J. W. , McClure, M. M. , Rogers, L. A. , & Schindler, D. E. (2010). Synchronization and portfolio performance of threatened salmon. Conservation Letters, 3, 340–348. 10.1111/j.1755-263X.2010.00119.x

[ece35530-bib-0030] Morris, J. F. T. , Trudel, M. , Thiess, M. E. , Sweeting, R. M. , Fisher, J. , Hinton, S. A. , … Welch, D. W. (2007). Stock‐specific migrations of juvenile coho Salmon derived from coded‐wire tag recoveries on the continental shelf of Western North America. American Fisheries Society Symposium, 57, 81–104.

[ece35530-bib-0031] Nilsson, J. (1990). Heritability estimates of growth‐related traits in Arctic charr (*Salvelinus alpinus*). Aquaculture, 84, 211–217. 10.1016/0044-8486(90)90087-4

[ece35530-bib-0032] Olsén, K. H. (1989). Sibling recognition in juvenile Arctic charr, *Salvelinus alpinus* (L.). Journal of Fish Biology, 34, 571–581. 10.1111/j.1095-8649.1989.tb03336.x

[ece35530-bib-0033] Quinn, T. P. , & Busack, C. A. (1985). Chemosensory recognition of siblings in juvenile coho salmon (*Oncorhynchus kisutch*). Animal Behaviour, 33, 51–56. 10.1016/S0003-3472(85)80119-6

[ece35530-bib-0034] Quinn, T. P. , & Hara, T. J. (1986). Sibling recognition and olfactory sensitivity in juvenile coho salmon (*Oncorhynchus kisutch*). Canadian Journal of Zoology, 64, 921–925.

[ece35530-bib-0035] R Core Team (2019). R: A language and environment for statistical computing. Vienna, Austria: R Foundation for Statistical Computing Retrieved from https://www.R-project.org/

[ece35530-bib-0036] Rohde, J. , Fresh, K. L. , & Quinn, T. P. (2014). Factors affecting partial migration in Puget Sound coho salmon. North American Journal of Fisheries Management, 34, 559–570.

[ece35530-bib-0037] Rohde, J. , Kagley, A. N. , Fresh, K. L. , Goetz, F. A. , & Quinn, T. P. (2013). Partial migration and diel movement patterns in Puget Sound coho salmon. Transactions of the American Fisheries Society, 142, 1615–1628. 10.1080/00028487.2013.822421

[ece35530-bib-0038] Sandercock, F. K. (1991). Life history of coho salmon (*Oncorhynchus kisutch*) In Groot C., & Margolis L. (Eds.), Life history of Pacific Salmon (pp. 395–406). Vancouver, BC: University of British Columbia Press.

[ece35530-bib-0039] Schindler, D. E. , Hilborn, R. , Chasco, B. , Boatright, C. P. , Quinn, T. P. , Rogers, L. A. , & Webster, M. S. (2010). Population diversity and the portfolio effect in an exploited species. Nature, 465, 609–612. 10.1038/nature09060 20520713

[ece35530-bib-0040] Steele, C. A. , Hess, M. , Narum, S. , & Campbell, M. (2019). Parentage‐based tagging: Reviewing the implementation of a new tool for an old problem. Fisheries, in press, 10.1002/fsh.10260

[ece35530-bib-0041] Tillotson, M. D. , & Quinn, T. P. (2018). Selection on the timing of migration and breeding: A neglected aspect of fishing‐induced evolution and trait change. Fish and Fisheries, 19, 170–181. 10.1111/faf.12248

[ece35530-bib-0042] Tipping, J. M. , & Busack, C. A. (2004). The effect of hatchery spawning protocols on coho salmon return timing in the Cowlitz River, Washington. North American Journal of Aquaculture, 66, 293–298. 10.1577/A04-007.1

[ece35530-bib-0043] Tucker, S. , Trudel, M. , Welch, D. W. , Candy, J. R. , Morris, J. F. T. , Thiess, M. E. , … Beacham, T. D. (2009). Seasonal stock‐specific migrations of juvenile Sockeye Salmon along the west coast of North America: Implications for growth. Transactions of the American Fisheries Society, 138, 1458–1480.

[ece35530-bib-0044] Tucker, S. , Trudel, M. , Welch, D. W. , Candy, J. R. , Morris, J. F. T. , Thiess, M. E. , … Beacham, T. D. (2012). Annual coastal migration of juvenile Chinook salmon: Static stock‐specific patterns in a highly dynamic ocean. Marine Ecology Progress Series, 449, 245–262. 10.3354/meps09528

[ece35530-bib-0045] Van Doornik, D. M. , Ford, M. J. , & Teel, D. J. (2002). Patterns of temporal genetic variation in coho salmon: Estimates of the effective proportion of 2‐year‐olds in natural and hatchery populations. Transactions of the American Fisheries Society, 131, 1007–1019. 10.1577/1548-8659(2002)131<1007:POTGVI>2.0.CO;2

[ece35530-bib-0046] Wang, J. (2016). Individual identification from genetic marker data: Developments and accuracy comparisons of methods. Molecular Ecology Research, 16, 163–175. 10.1111/1755-0998.12452 26230747

[ece35530-bib-0047] Weitkamp, L. (2011). Marine distributions of coho and Chinook Salmon inferred from coded wire tag recoveries. American Fisheries Society Symposium, 76, 1–23.

[ece35530-bib-0048] Weitkamp, L. , & Neely, K. (2002). Coho salmon (*Oncorhynchus kisutch*) ocean migration patterns: Insight from marine coded‐wire tag recoveries. Canadian Journal of Fisheries and Aquatic Sciences, 59, 1100–1115.

