# Supplementary material for: Variation in migration pattern, broodstock origin, and family productivity of coho salmon hatchery populations in British Columbia, Canada, derived from parentage‐based tagging
Source: Ecol Evol. 2019 Aug 18;9(17):9891–906. doi: 10.1002/ece3.5530 (PMC6745653; doi:10.1002/ece3.5530)
Supplement: Supplementary file 1 [file ECE3-9-9891-s001.docx]

Supplementary Table 1. Significant deviations from expected distributions within families of jack (and jill) PBT identifications restricted to individual brood males with at least four total PBT identifications inclusive of non-jack identifications. Npop is the number of males in the populations with four or greater total PBT identifications. n is the number of males in each population with the unique number of total PBT identifications and jack PBT identifications. P is the probability for listed male jack and non-jack PBT identifications determined by Fisher’s exact test.

| Population | Npop | Over contributions | | | |
| --- | --- | --- | --- | --- | --- |
|  |  | n | Total PBT | Jack PBT | P |
| Robertson | 78 | 1 | 6 | 3 | 0.0375 |
|  |  | 2 | 7 | 2 | 0.0403 |
|  |  | 1 | 13 | 7 | 0.0012 |
|  |  | 1 | 15 | 6 | 0.0081 |
|  |  | 1 | 19 | 8 | 0.0019 |
| Big Qualicum | 83 | 1 | 6 | 3 | 0.0217 |
|  |  | 1 | 7 | 3 | 0.0347 |
|  |  | 1 | 10 | 5 | 0.0029 |
| Quinsam | 120 | 2 | 4 | 2 | 0.0392 |
|  |  | 2 | 5 | 3 | 0.0056 |
|  |  | 1 | 6 | 4 | 0.0007 |
|  |  | 1 | 8 | 4 | 0.0029 |
|  |  | 1 | 8 | 3 | 0.0256 |
|  |  | 1 | 9 | 3 | 0.0359 |
|  |  | 1 | 10 | 7 | 0.0001 |
| Tenderfoot | 32 | 1 | 8 | 2 | 0.0284 |
| Capilano | 86 | 4 | 4 | 2 | 0.0217 |
|  |  | 1 | 9 | 3 | 0.0158 |
|  |  | 1 | 10 | 4 | 0.0025 |
| Chilliwack | 185 | 1 | 4 | 3 | 0.0053 |
|  |  | 1 | 5 | 4 | 0.0008 |
|  |  | 1 | 6 | 6 | 0.0001 |
|  |  | 1 | 6 | 5 | 0.0001 |
|  |  | 1 | 7 | 4 | 0.0044 |
|  |  | 2 | 7 | 3 | 0.0357 |
|  |  | 1 | 10 | 4 | 0.0198 |
|  |  | 1 | 14 | 6 | 0.0029 |
|  |  | 1 | 17 | 12 | 0.0001 |
| Inch | 51 | 1 | 7 | 4 | 0.0078 |
|  |  | 1 | 14 | 8 | 0.0002 |
|  |  | 1 | 19 | 9 | 0.0004 |
| Stave | 31 | 1 | 4 | 2 | 0.0243 |
|  |  | 1 | 6 | 5 | 0.0001 |
|  |  | 1 | 6 | 3 | 0.0053 |
| Chehalis | 47 | 1 | 6 | 3 | 0.0003 |

Supplementary Table 2. Significant deviations from expected distributions within families of fishery PBT identifications restricted to individual brood males with at least four total PBT identifications inclusive of both fishery and escapement identifications. Npop is the number of males in the populations with four or greater total PBT identifications. n is the number of males in each population with the unique number of total PBT identifications and fishery PBT identifications. P is the probability for listed male fishery and escapement PBT identifications determined by Fisher’s exact test.

| Population | Npop | Fishery | | | |
| --- | --- | --- | --- | --- | --- |
|  |  | n | Total PBT | Observed fishery PBT | P |
| Robertson | 78 | 1 | 5 | 5 | 0.0089 |
|  |  | 1 | 6 | 5 | 0.0358 |
|  |  | 3 | 7 | 6 | 0.0161 |
|  |  | 1 | 14 | 12 | 0.0005 |
| Conuma | 15 | 1 | 6 | 3 | 0.0329 |
| Nitinat | 37 | 1 | 4 | 2 | 0.0359 |
| Qualicum | 83 | 2 | 4 | 3 | 0.0062 |
| Quinsam | 120 | 1 | 5 | 3 | 0.0127 |
|  |  | 1 | 7 | 4 | 0.0046 |
| Capilano | 86 | 1 | 4 | 3 | 0.0453 |
|  |  | 3 | 6 | 4 | 0.0327 |
| Chilliwack | 185 | 1 | 6 | 5 | 0.0016 |
|  |  | 1 | 12 | 6 | 0.0193 |
| Inch | 51 | 1 | 5 | 5 | 0.0005 |
|  |  | 1 | 5 | 4 | 0.0098 |
|  |  | 1 | 10 | 6 | 0.0108 |
|  |  | 1 | 11 | 7 | 0.0038 |
| Norrish | 22 | 1 | 7 | 7 | 0.0138 |
| Stave | 31 | 1 | 5 | 2 | 0.0387 |
|  |  | 1 | 9 | 3 | 0.0190 |
| Chehalis | 47 | 1 | 5 | 3 | 0.0324 |

Supplementary Table 3. Significant deviations from expected distributions within families of escapement PBT identifications restricted to individual brood males with at least four total PBT identifications inclusive of both fishery and escapement identifications. Npop is the number of males in the populations with four or greater total PBT identifications. n is the number of males in each population with the unique number of total PBT identifications and escapement PBT identifications. P is the probability for listed male fishery and escapement PBT identifications determined by Fisher’s exact test.

| Population | Npop | Escapement | | | |
| --- | --- | --- | --- | --- | --- |
|  |  | n | Total PBT | Observed Escapement PBT | P |
| Robertson | 78 | 1 | 17 | 15 | 0.0236 |
|  |  | 1 | 23 | 19 | 0.0481 |
| Capilano | 86 | 1 | 15 | 15 | 0.0285 |
|  |  | 1 | 14 | 14 | 0.0493 |
| Chilliwack | 185 | 1 | 17 | 17 | 0.0332 |
| Inch | 51 | 1 | 21 | 21 | 0.0113 |
